# Tuberculous Meningitis: An Endemic Cause of Intracranial Hypertension

**DOI:** 10.7759/cureus.51532

**Published:** 2024-01-02

**Authors:** Miguel Costa, João Pedro Caria, João Bilardo Caiano, Alexandra Caeiro, Fernando Maltez

**Affiliations:** 1 Internal Medicine, Hospital Viana do Castelo, Viana do Castelo, PRT; 2 Infectious Diseases, Hospital Curry Cabral, Lisbon, PRT; 3 Neurology, Hospital Curry Cabral, Lisbon, PRT

**Keywords:** sixth cranial nerve palsy, increased intracranial pressure, presumptive diagnosis, mycobacterium tuberculosis, tuberculosis meningitis

## Abstract

Tuberculous meningitis (TBM) presents a complex clinical scenario, often marked by delayed recognition and high mortality. Our case involves a 27-year-old woman from Nepal with no significant medical history, presented with a two-week history of fatigue, altered consciousness, dizziness, vomiting, fever, holocranial headache, and photophobia. Initial examination revealed signs consistent with meningitis, including fever, hypertensive state, prostration, bilateral exophthalmos, sixth cranial nerve paresis, and positive Kernig/Brudzinski signs. Cerebrospinal fluid (CSF) exhibited characteristics typical of TBM: turbidity, lymphocytic-predominant pleocytosis, low glucose, and elevated protein. The patient was promptly started on meningeal doses of vancomycin, ceftriaxone, and acyclovir. However, persistent fever, neurological deterioration, and signs of increased intracranial pressure led to the decision to initiate conventional empiric treatment of tuberculosis (TB) with isoniazid, rifampicin, pyrazinamide, and ethambutol (HRZE) and dexamethasone 1 week before cultural positivity for *Mycobacterium tuberculosis* of CSF. The case underscores the importance of considering TBM in patients from endemic regions, interpreting CSF findings, and initiating empirical treatment in critical scenarios, contributing to a positive patient outcome despite the diagnostic challenges.

## Introduction

Tuberculous meningitis (TBM) is associated with high morbidity and mortality, particularly when there is a delay in diagnosis/treatment and HIV coinfection. Previous studies have reported a mortality of approximately 50% [1]. The incidence of TBM generally reflects the incidence and prevalence of tuberculosis in the community. In 2021, there were approximately 10.6 million cases of tuberculosis (TB) globally, with 1.6 million deaths among them. TBM is the most severe form of extrapulmonary tuberculosis, though it is less common (incidence, 5-15%). The highest incidence of TB worldwide is observed in Southeast Asia and Africa [2].

Diagnosing TBM is challenging due to nonspecific findings in clinical presentation. The initial differential diagnosis includes other bacterial, viral, or fungal infections of the central nervous system (CNS), non-infectious inflammatory diseases of the meninges (including systemic lupus erythematosus), and brain neoplasms (primary or metastatic) [3]. Patients with TBM typically exhibit meningitis symptoms such as headache, neck stiffness, and fever. However, meningeal signs may be absent in the early stages. Involvement outside the lungs can occur independently or along with a pulmonary focus, as seen in disseminated tuberculosis [4].

The inflammatory response induced by TBM is associated with various complications, including cerebrovascular disease, cranial nerve paralysis, and hydrocephalus [5]. Early diagnosis and prompt initiation of appropriate therapy are crucial to reduce high mortality and severe sequelae associated with the disease. The definitive diagnosis is established by the growth of *Mycobacterium tuberculosis* in a culture of cerebrospinal fluid (CSF) [6].

Risk factors for TBM include malnutrition, alcoholism, diabetes mellitus, immunodeficiency, chronic liver disease, etc. Its incidence has been primarily associated with defects in cell-mediated immune mechanisms, as seen in HIV-infected individuals, other immunosuppressive diseases, and even in individuals considered initially immunocompetent [4].

The preferred treatment for TBM is similar to the treatment used for pulmonary TB. Although rifampin penetrates the CSF less freely, its importance is confirmed by the higher mortality of TBM cases with rifampin-resistant strains [7]. The World Health Organization suggests that 9 to 12 months is sufficient for successful TBM treatment. Usually, two months of daily isoniazid, rifampin, pyrazinamide, and ethambutol (HRZE) are followed by 7-10 months of isoniazid and rifampin (HR) [8]. Adjunctive corticosteroids should be given in patients with TBM, as evidence suggests a mortality benefit. Dexamethasone exerts its beneficial effect by inhibiting the synthesis of inflammatory cytokines, decreasing CSF outflow resistance, and stabilizing the blood-brain barrier [9].

## Case presentation

**Figure 1:**
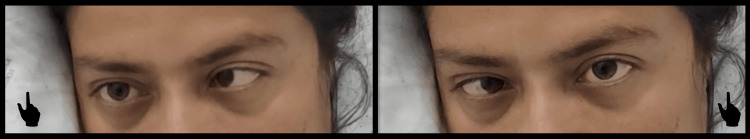
Sixth cranial (abducens) nerve palsy Sixth cranial nerve palsy affects the lateral rectus muscle, impairing eye abduction. The patient showed a bilateral inability to perform ocular abduction, confirming the presence of bilateral paresis of the sixth cranial nerve. The hand symbol represents the focal point determined by the physician toward which the patient is attempting to direct their gaze.

A 27-year-old woman from Nepal, with no significant medical history, presented to the emergency department with fatigue, altered consciousness, dizziness, vomiting, fever, holocranial headache, and photophobia over two weeks. On examination: febrile (38.4ºC), slightly hypertensive (164/95 mmHg), prostrate but responsive to verbal stimuli with active mobilization of all 4 limbs (GCS: 13), bilateral exophthalmos, paralysis of the sixth cranial nerve, neck stiffness, and positive Kernig/Brudzinski signs. Figure 1 shows part of the patient's neurologic examination where sixth cranial nerve palsy is evident.

Laboratory tests showed no significant inflammatory parameters and a mild hyponatremia with increased urinary sodium. Viral serologies and venereal disease research laboratory (VDRL) tests were negative. Contrast-enhanced CT scan without abnormalities. Chest CT scan without pleuroparenchymal changes. Lumbar puncture (LP) was performed, which showed a turbid cerebrospinal fluid (CSF), and its analysis revealed a pleocytosis with lymphocyte predominance (81%), low glucose, elevated protein, and a normal level of adenosine deaminase (ADA). The nucleic acid amplification test (NAAT) for mycobacteria was also immediately tested in CSF and was negative. CSF sent for CRP of neurotropic viruses, bacteriological, and mycobacteriological cultures. Table 1 schematically shows in detail the analyses at admission.

**Table 1:** Blood, CSF, and urine analysis on admission. Despite being febrile and having cerebrospinal fluid compatible (CSF) with central nervous system infection (lymphocytic-predominant pleocytosis, hypoglycorrhachia, and elevated protein), analytically, the patient did not show a significant increase in inflammatory parameters (without leukocytosis, and CPR only minimally elevated). Note that, the PCR virus panel was only revealed a few days later. The range of values within parentheses refers to the normal expected values for a young adult female. A mild hyponatremia was found in association with urine sodium >30 mmol/l, which suggests renal sodium loss or SIADH. Furthermore, a urine osmolarity >100mOsmol/Kg in the presence of low blood osmolarity is also one criterion for SIADH. ADA: Adenosine deaminase; CMV: Cytomegalovirus; CPR: C-reactive protein; CSF: Cerebrospinal fluid; EBV: Epstein-Barr virus; HBV: Hepatitis B virus; HCV: Hepatitis C virus; HIV: Human immunodeficiency virus; HHV: Human herpes virus; HSV: Herpes simplex virus; INR: International Normalized Ratio; LDH: Lactate dehydrogenase; PCR: polymerase chain reaction; SIADH: Syndrome of inappropriate antidiuretic hormone (ADH) release; VDRL: Venereal disease research laboratory test (screening test for syphilis).

Laboratory data	Results
Hemoglobin (12 to 16 g/dl)	13.5 g/dL
Leucocytes (<10.000/mm^3^)	9.440/mm^3^
Platelets (150,000 to 450,000/mm^3^)	262.000/mm^3^
INR (<1.1)	0.98
LDH (140 to 280 U/L)	284 UI/L
Glucose (70-140 mg/dL)	123 mg/dL
Sodium [Na+] (135-145 mmol/L)	130 mmol/L
Potassium [K+] (3.5-5.5 mmol/L)	3.7 mmol/L
Urea [Ur] (<50mg/dL)	45 mg/dL
Creatinine [Cr] (<1.2 mg/dL)	0.7 mg/dL
CPR (<0.5 mg/dL)	1 mg/dL
VDRL	Negative
CSF [cytologic] (Leucocytes <5/mm^3^; Erythrocytes <5/mm^3^)	Leucocytes 483/mm^3 ^with 394 Lymphocytes (81%); Erythrocytes 14/mm^3^
CSF [chemistry] (Glucose 50-80mg/dL; Proteins 15-60 mg; ADA <10 UI/L)	Glucose 10 mg/dL; Proteins 107 mg/dL; ADA 7.9 UI/L
CSF (PCR virus panel): HSV 1 and 2, CMV, EBV, HSV, HHV-3 and Enterovirus	Negative
PCR for COVID-19 (SARS-CoV-2)	Negative
Urine sediment	No alterations
Sodium in urine	48 mmol/L
Urine osmolarity	530 mOsmol/Kg
Blood osmolarity (275-295 mOsmol/Kg)	270 mOsmol/Kg
Virus serology HBV, HCV, HIV 1-2, CMV, and EBV	Negative

**Figure 2:**
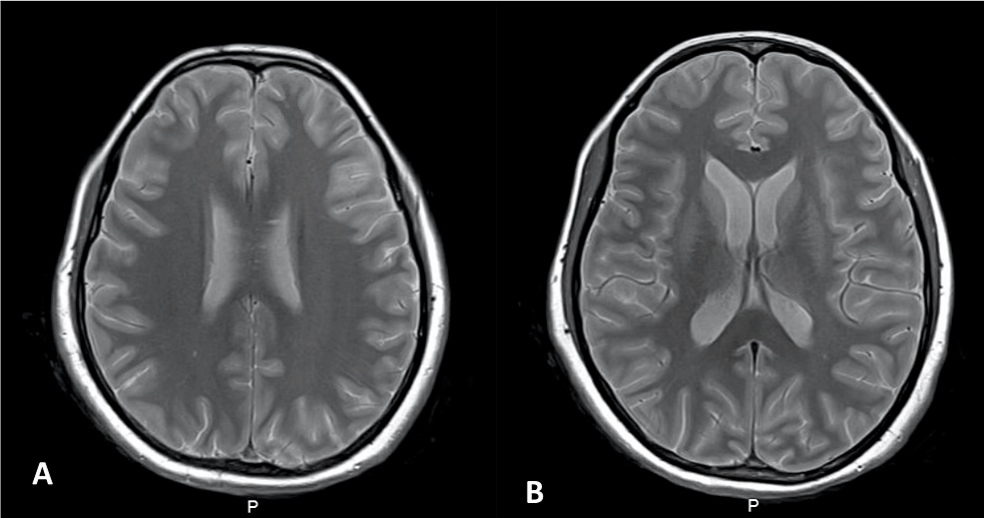
Cranial MRI scan on the fourth day of hospitalization in different cuts (images A and B). Both images reveal T2 hyperintensity in some sulci, leptomeningeal enhancement, and ventricular enlargement (more visible in image B).

The patient was hospitalized with meningeal doses of vancomycin, ceftriaxone, and acyclovir. Despite therapy initiated on the fourth day of admission, she continued to have a fever, neurological deterioration, and signs of increased intracranial pressure (high blood pressure, papilledema, headache, and diplopia). Repeat LP: worsened lymphocytosis and opening pressure of 36 cm of water. Considering viral meningoencephalitis or TBM as the most likely diagnosis, anti-tuberculosis drugs (conventional empiric treatment with HRZE) and dexamethasone were initiated at this point while continuing acyclovir. A cranial MRI scan at this time revealed T2 hyperintensity in some sulci, leptomeningeal enhancement, and ventricular enlargement (Figure 2A, 2B).

From the tenth day (after 5 days of anti-tuberculosis drugs) with improvement in general condition, regression of signs of increased intracranial pressure, and sustained afebrile state. Only on the twenty-fifth day, the *Mycobacterium tuberculosis* complex was isolated in CSF culture, confirming TBM and secondary hyponatremia. The patient was discharged asymptomatic and without any neurological deficits, under anti-tuberculosis drugs (to complete 2 months of a 4-drug regimen with HRZE, followed by 9 months of a 2-drug regimen with HR) and corticosteroid tapering.

## Discussion

Due to similarities with other diseases, slow growth of *Mycobacterium tuberculosis* in culture (1-8 weeks), and lower cultural confirmation rates in extrapulmonary tuberculosis, delayed diagnosis of TBM is common, leading to increased mortality rates [3]. Some studies suggest that a delay in treatment initiation of more than 3 days after admission is associated with a 70% increase in mortality [1]. Therefore, if the clinical presentation, CSF findings, and neuroimaging studies of a patient with meningitis are consistent with TBM, and other etiologies are excluded through initial CSF analysis, a presumptive diagnosis of TBM should be made, and treatment should be promptly initiated [10].

On the fourth day of hospitalization, with continued fever and neurological deterioration, therapeutic failure was considered. Note that ceftriaxone, vancomycin, and acyclovir were administered from the beginning at meningeal doses. At this point, bacteriological and mycobacteriological cultures of CSF were still ongoing without growth. The possibility of meningoencephalitis due to typical bacterial agents was ruled out. The negativity of NAAT for *Mycobacterium tuberculosis* was already known; however, in non-bacillary patients, the sensitivity of these tests is less than 50% [11].

In addition to the lack of response to conventional antibacterial agents for meningitis, several other clues pointed towards TBM as a strong diagnostic hypothesis: (1) Patient's origin from an endemic TB region [1]; (2) Characteristic CSF findings favoring the diagnosis of TBM: CSF findings of TBM include a lymphocytic-predominant pleocytosis, elevated protein (typically between 100 and 500 mg/dL), and low glucose (usually less than 45 mg/dL or CSF: plasma ratio <0.5) [7]; (3) Presence of signs of increased intracranial pressure (ICP): Elevated ICP is common in TBM patients. There are no systematic studies to estimate the incidence of elevated ICP in patients with TBM, but in the cases described in the bibliography, most of the patients with severe illness are likely to have elevated ICP. Note that in this patient, arterial hypertension was also an indirect sign of increased intracranial pressure (ICP) and a self-protective mechanism for the patient to maintain adequate hypoperfusion. The paresis of the sixth cranial nerve would be another indirect sign of increased intracranial pressure. In fact, considering its length and location, the abducent nerve (VI) is the first to be affected by compression secondary to increased intracranial pressure. TBM displays secondary brain injury because of the inflammatory reaction induced by the mycobacteria released, leading to a local T-cell-dependent response. This cascade results in the breakdown of the blood-brain barrier, disruption of cerebral autoregulation, accumulation of excitatory amino acids and free radicals, the cellular inflammatory response, and regional hyperthermia. The ultimate outcome of secondary brain injury is cytotoxic and vasogenic edema, elevated ICP, and cell death [12]; (4) Presence of euvolemic hyponatremia with slightly increased urinary Na+ and elevated urinary osmolality, favoring the diagnosis of syndrome of inappropriate antidiuretic hormone (SIADH): Hyponatremia has long been recognized as a potentially serious metabolic consequence of TBM occurring in 35-65% of the cases. The SIADH secretion has long been believed to be responsible for the majority of cases of hyponatremia in TBM [13].

Among the mentioned clues, epidemiology and lymphocytic-predominant pleocytosis in CSF would have higher specificity for TBM. Given the neurological status and the possibility of TBM, without empirical therapy, the patient likely would have died or suffered irreversible neurological consequences before confirming the diagnosis through culture. Therefore, on the fourth day, it was decided to initiate anti-tuberculosis drugs and dexamethasone, discontinuing previous antibiotic therapy and maintaining acyclovir.

Unlike other bacterial meningitis, TBM may present with normal or only slightly elevated PCR values [14]. In fact, the patient consistently had relatively low levels of CPR (1-5mg/dL) from the beginning. The spacing of fever peaks, improvement of hyponatremia, neurological status, and reduction in blood pressure values, as an indirect measure of improved ICP, was essential to consider a favorable response to anti-tuberculosis drugs within the first 48 hours after a change in therapeutic strategy. On the tenth day, she objectively improved in general condition, completely regressed signs of increased ICP, and maintained sustained afebrility. At this point, acyclovir was discontinued, especially since the entire virus PCR panel was negative. Given the patient's status on the fourth day (severe ICP, prostration, sixth cranial nerve paresis, and persistent fever) when anti-tuberculosis drugs were initiated and the time needed for cultural positivity of CSF (25 days), the favorable vital and functional prognosis of the patient (discharged autonomously without any neurological deficits) would not have been possible without the empirical initiation of HRZE therapy assuming TBM as a presumptive diagnosis.

## Conclusions

The high morbidity and mortality of TBM are directly related to the delay in initiating appropriate treatment. Since the clinical presentation is nonspecific and the sensitivity of NAAT for mycobacteria is low, the definitive diagnosis depends on the time-consuming cultural examination. In some cases, it is essential to precede the start of treatment based on a presumptive diagnosis. In this case, the high suspicion due to the presence of increased intracranial pressure, origin from an endemic region, lymphocyte predominance in CSF with hypoglycorrhachia, and lack of response to conventional antibacterials was crucial for the timely initiation of targeted therapy and a positive outcome for the patient.
